# The Effect of a Kasai Procedure on Liver Transplantation in Children with Biliary Atresia: A Cohort Study

**DOI:** 10.3390/jcm14103328

**Published:** 2025-05-10

**Authors:** Hao Dong, Jing-Yi Liu, Yi-Zhou Jiang, Li-Ying Sun, You-Xin Wang

**Affiliations:** 1 Beijing Key Laboratory of Clinical Epidemiology, School of Public Health, Capital Medical University, Beijing 100069, China; donghao8911@mail.ccmu.edu.cn; 2 Department of Critical Liver Diseases, Liver Research Center, Beijing Friendship Hospital, Capital Medical University, Beijing 101100, China; 112020010322@mail.ccmu.edu.cn (J.-Y.L.); jiangyizhou@zryhyy.com.cn (Y.-Z.J.); 3 Liver Transplantation Center, National Clinical Research Center for Digestive Diseases, Beijing Friendship Hospital, Capital Medical University, Beijing 101100, China; 4 Clinical Center for Pediatric Liver Transplantation, Capital Medical University, Beijing 100050, China; 5 Department of Infectious Disease, China-Japan Friendship Hospital, Beijing 100029, China; 6 School of Public Health, North China University of Science and Technology, Tangshan 063210, China

**Keywords:** biliary atresia, liver transplantation, outcome, Kasai hepatoportoenterostomy, nomogram

## Abstract

**Background/Objectives**: Biliary atresia (BA) is the most common etiology for pediatric liver transplantation (LT). However, whether a previous Kasai hepatoportoenterostomy (KP) and its timing influence the outcomes of BA patients who undergo LT remains controversial. **Methods**: Pediatric patients with BA who underwent LT at Beijing Friendship Hospital, Capital Medical University, between June 2013 and November 2022 were recruited. The patients were divided into non-KP, early-KP (before 90 days of life), and late-KP subgroups. The clinical data were compared among the groups. A nomogram to predict the 1-, 3-, and 5-year graft survival probabilities based on a multivariate Cox model was constructed and validated. **Results**: Among the 475 BA patients, the no-KP group accounted for 31.8%, the early KP for 60.4%, and the late KP for 7.8%, respectively. The incidences of LT complications were comparable among the groups. From the multivariate Cox analyses, an intensive care unit (ICU) stay and bleeding were identified as the independent risk factors for postoperative patient survival, and the LT type, graft type, vascular complications, and biliary complications were those for graft survival. A nomogram for graft survival was constructed, with a C-index of 0.82, and areas under the curves (AUCs) of 0.829, 0.824, and 0.824 for the 1-, 3-, and 5-year survival nomograms, respectively. The calibration and decision curve analysis (DCA) curves showed good discrimination ability and clinical applicability. A risk classification system was further developed, and the Kaplan–Meier curves demonstrated high discrimination between the high- and low-risk groups (*p* < 0.0001). **Conclusions**: A previous KP has no impact on patients or graft survival after LT in BA patients. The established nomogram may be helpful for counseling BA patients about their clinical prognosis after LT.

## 1. Introduction

Biliary atresia (BA) is a severe cholestatic disease involving the intrahepatic and extrahepatic bile ducts, which is the most common cause of obstructive jaundice in infants and the leading cause of pediatric cholestatic liver disease [1]. A Kasai hepatoportoenterostomy (KP), namely a Roux-en-Y hepatic portoenterostomy procedure, is the current standard corrective treatment to save the lives and improve the prognoses of children with BA [2]. Notably, although a successful Kasai procedure can salvage the native liver and reestablish biliary flow, about 50% of patients with BA experience failure of the bile drainage surgery, and eventually receive a liver transplantation (LT) or die. Therefore, a timely Kasai procedure, ideally performed by the age of 2 months, has been considered to be crucial. When delayed beyond 4 months of age, the hepatic portoenterostomy procedure achieves successful bile drainage in merely 7% of cases. Moreover, those who live with their native livers still suffer from many long-term postoperative complications, such as recurrent cholangitis, portal hypertension, ascites, infections, gastrointestinal bleeding, and failure to thrive [3].

Since the first attempt in 1963, LT has become an established therapeutic option for patients with BA who cannot achieve successful bile drainage via a Kasai procedure or who suffer from highly advanced postoperative liver sequelae [4,5]. BA is also the most common indication for pediatric LT worldwide [5]. However, the role of LT as a primary procedure in BA has been argued over for at least 40 years [6,7,8]. More importantly, the impact of a previous Kasai procedure, as well as its timing, on the clinical outcome of an LT remains debatable. Thus, some scholars have questioned the necessity of a Kasai procedure [9,10]. Some previous studies have found that a previous Kasai procedure may increase the risk of intraoperative bleeding, biliary complications, and intestinal perforation. In contrast, other studies have showed that a previous Kasai procedure can delay the timing of an LT, prolonging the age and increasing the body weight at the time of the LT, and improve the preoperative status of children with BA [9].

Here, as the largest single-center report on pediatric LT for BA patients, we aim to provide clinical evidence by comparing the liver transplant outcomes in patients with BA who received an early KP (patients with Kasai procedure performed before 90 days of life), late KP (patients with Kasai procedure performed after 90 days of life), and no KP (patients without a Kasai procedure who were referred for primary LT).

## 2. Materials and Methods

### 2.1. Study Population

This is a single-center retrospective cohort study. All consecutive pediatric liver recipients who underwent LT with the diagnosis of BA at the Liver Transplantation Center of Beijing Friendship Hospital, Capital Medical University, between Jun 2013 and November 2022 were enrolled in this study. The exclusion criteria included the following: (1) explant livers that were not supportive of a diagnosis of BA, (2) Patients combined with an etiology of progressive familial intrahepatic cholestasis or Alagille syndrome, (3) re-transplantation, and (4) those lost to follow-up or whose detailed clinical information was unavailable. No organs were obtained from executed prisoners. This study was conducted under the ethical guidelines of the 1975 Declaration of Helsinki and was approved by the Ethics Committee of Beijing Friendship Hospital.

Patients were divided into the no-KP group (without a Kasai procedure who were referred for primary LT) and the KP group. Two subgroups within the KP group were further defined as follows: early KP (patients with Kasai procedure performed before 90 days of life) and late KP (patients with Kasai procedure performed after 90 days of life).

**Figure 1:**
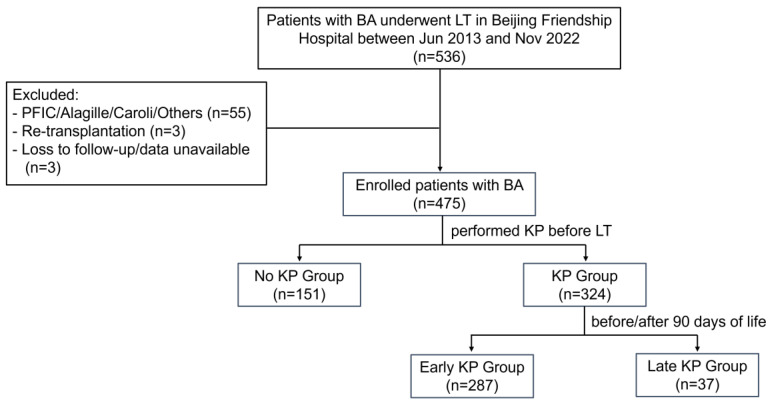
Flowchart for the enrolled patients with biliary atresia in this study. BA, biliary atresia; KP, Kasai hepatoportoenterostomy; LT, liver transplantation; PFIC, progressive familial intrahepatic cholestasis.

During the study period, 536 pediatric patients with BA underwent LT at our institution. Of these, 55 patients were diagnosed with progressive familial intrahepatic cholestasis, Alagille syndrome, Caroli’s disease, and other cholestatic diseases based on the pathological findings of the resected diseased liver; 3 patients received liver re-transplantation; and 3 patients had incomplete data and were excluded. Finally, 475 patients were included in this retrospective study (Figure 1).

### 2.2. Clinical Data and Follow-Up

Data were extracted from the patient database of our hospital. The baseline demographic and LT data, including sex; age at KP and LT; donor type; graft type; GRWR (graft-to-recipient weight ratio); cold ischemic time (CIT, defined as the interval from initiation of donor in vivo cold organ preservation to removal of the graft from 4 °C cold storage); warm ischemic time (WIT, defined as the interval from the cessation of perfusion of the graft to the initiation of cold preservation); intraoperative blood loss; operation time; duration of mechanical ventilation; length of ICU stay; length of hospital stay; routine laboratory data, including the values of alanine aminotransferase (ALT), aspartate aminotransferase (AST), gamma-glutamyl transpeptidase (GGT), and total bilirubin (TB); and imaging results before LT were collected. Patients were followed up with for at least 6 months. In our center, immunosuppressive regimens mainly consist of a calcineurin inhibitor and steroid [11]. Liver function tests, drug trough level, and abdominal ultrasonography were assessed as per routine follow-up protocol [12]. Closer check-ups and additional examinations were performed when patient status required.

### 2.3. Outcomes

The main outcomes of this study were graft and patient overall survival rates, calculated according to the Kaplan–Meier estimates. Additional outcomes included postoperative morbidity from biliary complications (leakage, stenosis, etc.) and vascular complications (portal vein stenosis, hepatic artery thrombosis, etc.).

### 2.4. Statistical Analysis

For continuous variables, a Kolmogorov–Smirnov normality test should first be performed. Means and standard deviations (SDs) were calculated for normally distributed data, and medians and interquartile ranges (IQRs) for non-normally distributed data. To determine the statistical significance of differences among the three subgroups, we employed an ANOVA test for normally distributed data, which was subsequently complemented by a post hoc Tukey–Kramer test for multiple comparisons. For data that did not adhere to normal distribution, we utilized a Kruskal–Wallis test, followed by a post hoc Bonferroni test for multiple comparisons. A chi-square test and Fisher’s exact test were used for categorical variables.

Univariate and multivariate Cox regression analyses were used to identify the independent risk factors and build the clinical models. Only variables demonstrating statistical significance (*p* < 0.05) in univariate analysis were included in the multivariate regression model. Furthermore, a nomogram for post-LT graft survival was established based on the multivariate Cox model. C-index and receiver operating characteristic (ROC) curves were calculated to evaluate the accuracy of the model. Calibration curves (three groups, 100 bootstrap resamples) and decision curve analysis (DCA) were applied to validate the discrimination and clinical applicability of the constructed nomogram (by enabling individualized, multivariable risk prediction with dynamic visualization, enhancing clinical precision and adaptability in complex patient management). Finally, the patients were stratified into low-risk and high-risk groups based on the cutoff value of total points on the nomogram. The Kaplan–Meier estimates and the Mantel–Cox log-rank test were assessed to compare the graft survival differences between groups. The data collected in this study were analyzed using the statistical software SPSS 24 (IBM SPSS, Inc., Armonk, NY, USA) and R version 4.0.3. Only a two-tailed *p*-value less than 0.05 was considered statistically significant.

## 3. Results

### 3.1. Patient Characteristics

The demographics and laboratory data of the recipients and donors are summarized in Table 1. A total of 151 patients (31.8%) in the no-KP group had an overall mean age at LT of 7.7 months (6.2–9.5 months) and included 71 male patients (47.0%), whereas the 287 patients (60.4%) who underwent a Kasai procedure within 90 days of life were the early-KP failure group, and their overall average age at LT was 15.0 months (8.5–46.9 months) and the group included 145 male patients (50.5%). In contrast, the remaining 37 patients (7.8%) who underwent a Kasai procedure performed after 3 months of life were considered the late-KP group. A comparison of the preoperative data among the three groups showed that there were no significant differences in sex; preoperative liver function indexes (ALT, AST, and GGT); and routine blood investigations, including white blood cell (WBC) count, hemoglobin (HB), and platelet counts (Table 1). The median weight at LT in the no-KP group was significantly lower than for those in the early-KP and late-KP groups (7.3 [6.5–8.2] vs. 9.3 [7.0–15.5] and 8.5 [6.7–16.0] kg; *p* < 0.001). Undoubtedly, the median age at LT in the no-KP group was significantly younger than those in the early-KP and late-KP groups (7.7 [6.2–9.5] vs. 15.0 [8.5–46.9] and 9.9 [7.8–56.4] months; *p* < 0.001). The cholestatic parameter comparison showed statistically higher TBIL (medians: 167.0 vs. 72.7 and 118.6 µmol/L; *p* < 0.001) levels in the no-KP group.

More patients in the no-KP group (83.4%) received LDLT than those in the early-KP and late-KP groups (68.6% and 62.2; *p* = 0.001). Similarly, the GRWR was also significantly higher in the no-KP group (3.4 [3.0–4.0]) than in the early-KP (2.8 [2.1–3.6]) and late-KP (3.1 [2.1–4.0]) groups (*p* < 0.001). There was no significant difference in the warm ischemia time, operation time, intraoperative blood loss, or hospitalization among these groups. However, the cold ischemia time in the no-KP group was significantly lower than that in the early-KP and late-KP groups (1.4 [1.1–2.1] vs. 2.0 [1.4–6.0] and 2.2 [1.85–6.3] hours; *p* < 0.001) (Table 2). The mean follow-up periods for the no-Kasai, early-Kasai, and late-Kasai groups were 6.7, 5.2, and 5.2 years, respectively. As for the main complications after LT, the data in Table 2 show that the incidence of vascular complications, re-transplantation, and bleeding did not differ significantly among the three groups. However, there was a trend of a lower incidence of biliary complications in the no-KP group (2.0%) than in those in the early-KP (6.6%) and late-KP (10.8) groups (*p* = 0.03). Similarly, the risk of infection after LT was significantly lower in the patients with no KP than those with an early Kasai (18.5 versus 32.1; *p* = 0.01).

### 3.2. Identification of Predictive Risk Factors Using the Cox Model

According to the results of the univariate Cox regression analysis, we found that for the KP group, the AST level, GGT level, neutrophils %, GRWR, graft type, operation time, ICU stay, vascular complications, and bleeding were associated with patient mortality (*p* < 0.05). Finally, the ICU stay [hazard ratio (HR) = 1.00; 95% confidence interval (CI): 1.00–1.00; *p* = 0.002] and bleeding (HR = 5.37; 95% CI: 1.58–18.24; *p* = 0.007) were identified as the independent risk factors for postoperative patient mortality in the further multivariate analyses (Supplementary Table S1). In addition, the univariate analyses indicated that the KP group, sex, LT type, graft type, operation time, intraoperative blood loss, vascular complications, and biliary complications were significantly correlated with post-transplant graft loss (*p* < 0.05). These potential risk factors were evaluated through a multivariate regression analysis, which indicated that DDLTs (HR = 6.20; 95% CI: 2.22–17.38; *p* < 0.001), graft types other than whole liver and left lateral segments (HR = 2.03; 95% CI: 1.11–3.72; *p* = 0.02), vascular complications (HR = 5.60; 95% CI: 1.78–17.60; *p* = 0.003), and biliary complications (HR = 4.55; 95% CI: 1.22–16.95; *p* = 0.02) were the independent risk factors (Supplementary Table S2).

### 3.3. Nomogram Development and Validation

**Figure 2:**
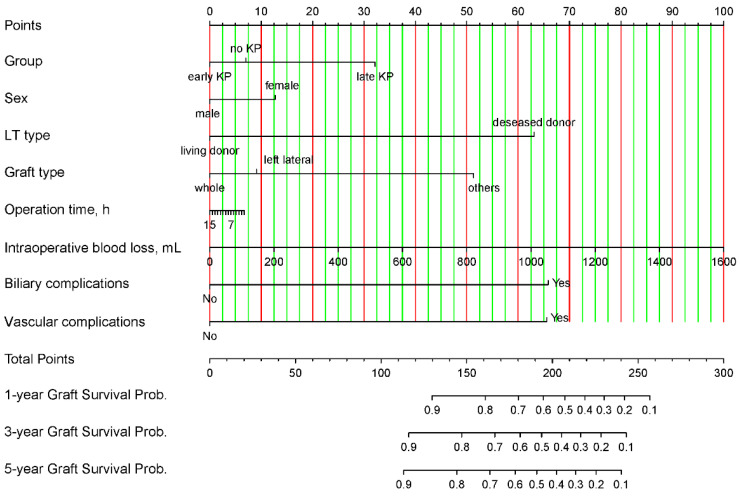
Nomogram for predicting the 1-, 3-, and 5-year graft survival probabilities in patients with biliary atresia. LT, liver transplantation; KP, Kasai hepatoportoenterostomy.

Based on the independent risk factors for post-transplant graft loss derived from the above Cox proportional hazard regression analyses, a nomogram was developed to predict the 1-, 3-, and 5-year graft survival probabilities, respectively (Figure 2). The total nomogram score was calculated as the sum of the scores for each of the factors.

**Figure 3:**
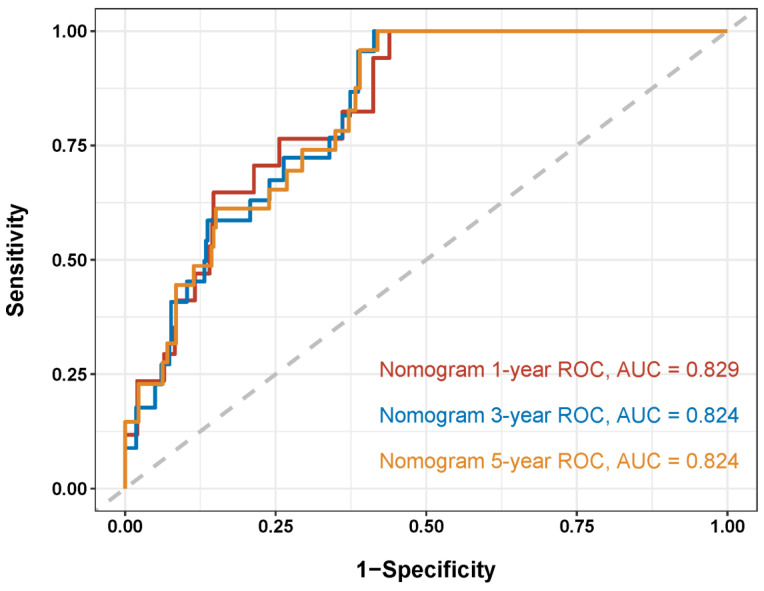
Receiver operating characteristic (ROC) curves for the nomograms for the 1- (red), 3- (blue), and 5-year (yellow) graft survivals.

**Figure 4:**
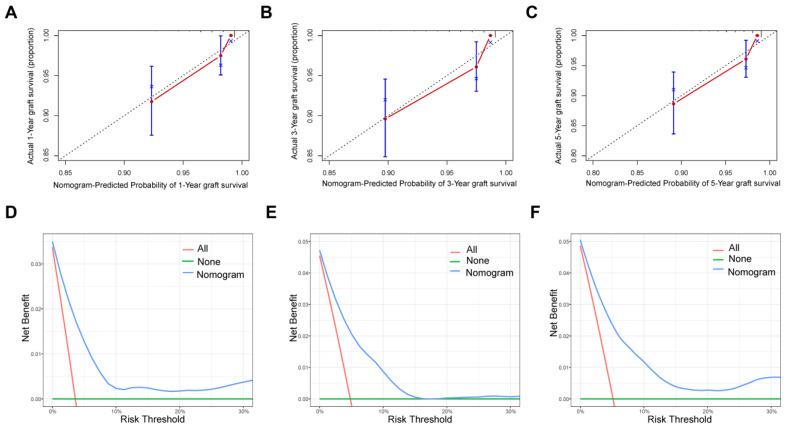
Calibration curves for the nomograms for the 1- (**A**), 3- (**B**), and 5-year (**C**) graft survivals of the cohort. The 45-degree black dashed line represents the perfect match between the actual (*y*-axis) and nomogram-predicted (*x*-axis) graft survival probabilities (bootstrap = 1000). The decision curve analysis for the nomograms for the 1- (**D**), 3- (**E**), and 5-year (**F**) graft survivals of the cohort. The y-axis represents the net benefit, whereas the x-axis represents the risk threshold.

To analyze the feasibility and validity of the new model, we tested the prognostic performance of the novel nomogram by applying AUC, calibration curves, and decision Curve Analysis (DCA) analyses. The overall C-index of the model at different follow-up times was 0.82. As shown in the ROC curves for the nomogram prediction models, the 1-, 3-, and 5-year AUCs for the nomogram of graft survival were 0.829, 0.824, and 0.824, respectively (Figure 3). These indicate the good discrimination ability of the nomogram model. The calibration curves also reveal good consistency between the actual observations and the nomogram predictions of the 1-, 3-, and 5-year graft survivals (Figure 4A–C), demonstrating the superior discriminative power of the model. In addition, we utilized DCA curves to verify the clinical applicability of the nomogram. As shown in Figure 4D–F, the DCA shows the good positive net benefits of the nomogram with different threshold probabilities, indicating it is suited for estimating individual survival.

### 3.4. Risk Classification

**Figure 5:**
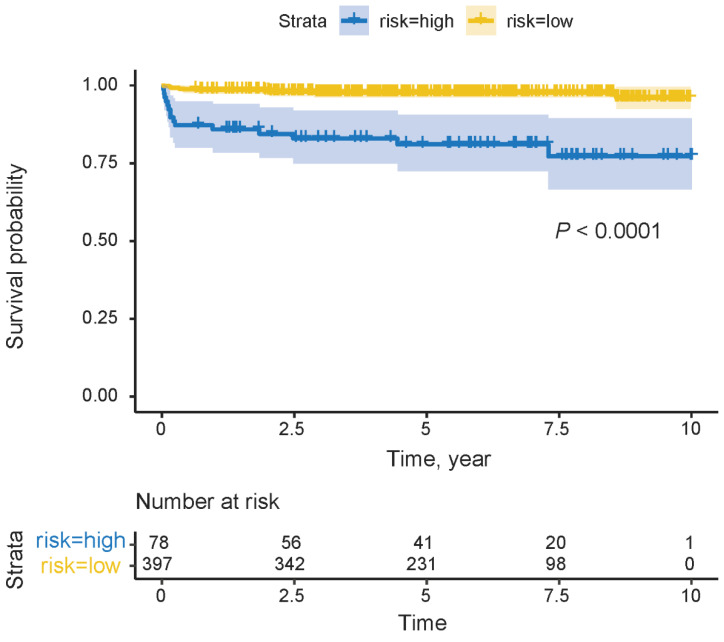
Kaplan–Meier curves of graft survival between low-risk (in yellow) and high-risk (in blue) groups, stratified by cutoff value for cohort.

Based on the total points of the nomogram, a risk classification system for graft survival was developed. The patients were divided into high-risk (78/475, 16.4%) and low-risk (397/475, 83.6%) groups according to the cutoff value (score of 100). The Kaplan–Meier curves showed the cumulative graft survival possibility of the high-risk groups was significantly lower than that of the low-risk groups (*p* < 0.0001), which demonstrates the high discrimination of the risk classification system (Figure 5).

## 4. Discussion

A KP is the procedure that is often used to extend the hepatic lifespan of patients with BA. Although most BA patients may eventually inevitably receive a liver transplant, Kasai surgery has been shown to significantly delay the timing of LT and improve the preoperative status of children [9]. However, some studies have argued that this surgical procedure may increase the risk of intraoperative bleeding and post-LT surgical complications, and since patients will eventually receive a liver transplant, in this era of rapid advances in pediatric LT technology, why not just skip Kasai surgery and go straight to a liver transplant? In this study, we demonstrated that for children with BA undergoing LT, although a previous Kasai procedure, either early or late, may increase the risk of biliary complications and infections, it has no significant influence on the patient’s or graft’s survival. With the largest single-center cohort of BA patients who underwent a pediatric LT, this study also developed and validated a nomogram that, by integrating the demographic data and clinical information, can be conveniently used to accurately predict the 1-, 3-, and 5-year graft survival probabilities of BA patients. Among the prognostic factors, a deceased donor and vascular and biliary complications are the largest contributors. Our prognostic nomogram shows good performance and can be an accurate tool to predict the individualized survival time of BA patients.

Living donor liver transplantation (LDLT), which can provide a large pool of organs, has emerged as an excellent alternative to deceased donor liver transplantation (DDLT) for patients with end-stage liver disease in the current circumstance of a shortage of deceased donors [13,14]. By expanding the donor pool and reducing waitlist mortality, a living donor has become the most common donor type for pediatric LT, especially in Asian countries where access to deceased organ donation is limited. In our study, the proportion of LDLT was 72.8%, nearly three times higher than DDLT. Previous studies have suggested that living donor recipients have better graft survival compared with deceased donor recipients among pediatric patients [15,16,17], with a 5-year graft survival of 91.2% compared with 84.8% in the United States [16]. Among BA patients, LDLT has also been associated with superior patient and graft survival outcomes than those of deceased or split deceased LT [5,18]. In our multivariate analysis, after adjusting for the confounding factors, we also found that a deceased donor was a significant risk factor for reduced graft survival in BA patients after LT. In addition, it has been reported that a deceased technical variant vs. whole graft (HR = 1.963) is a risk factor for graft loss [19]. The reasons for the optimal graft outcomes of LDLTs might include a shorter waitlist time, younger donor age, less ischemia time, improved donor selection, and controllable surgical procedures [5,17]. Since it provides an excellent alternative to DDLT without compromising recipient outcomes, LDLT remains a valuable therapeutic option for patients with BA in need of an LT [18,20].

Post-transplant complications are a dominant risk factor for patient mortality, and a recent single-center retrospective cohort study identified that an upper respiratory tract infection, hemorrhage, and intestinal perforation were three major risk factors that were associated with patient mortality after a pediatric LT [21]. The duration of the ICU stay has been found to be independently associated with early mortality after an adult LDLT [22]. Similarly, our study found that a prolonged ICU stay and postoperative bleeding complications were independent risk factors for patient mortality following a LT. A prolonged ICU stay may reflect poor postoperative recovery or severe physiological disturbances in patients. Postoperative bleeding, on the other hand, is a serious complication of an LT that can lead to hemodynamic instability and multiple organ failure. Comprehensive preoperative assessments (e.g., MELD score, nutritional status evaluation) should be conducted to identify high-risk patients and develop individualized treatment plans. Additionally, close postoperative monitoring of hemoglobin levels, coagulation function, and abdominal drainage is crucial for the early detection of bleeding and timely intervention. Optimizing postoperative management through collaboration among the LT team, ICU team, and nutritional support team is also vital.

Postoperative vascular complications, including hepatic artery thrombosis (HAT) and portal vein thrombosis (PVT), are serious complications after LT, which have been shown to represent the second most common cause of liver graft failure, after graft rejection, being associated with increased overall mortality among pediatric LT recipients [18,23]. Thus, reducing the vascular complication rates could provide superior long-term survival outcomes of pediatric LTs [23]. Consistent with the current literature, our multivariate regression analysis also found that vascular complications are indeed the independent risk factors for post-transplant graft loss. Notably, compared with adult recipients, liver transplants in pediatric recipients have always increased the risk of vascular complications, owing to the smaller vascular diameters [24]. In this scenario, increasing the experience and proficiency of the doctors, making technical/protocol modifications, early diagnosis, and the adaptation of a multimodality treatment, are the keys to decreasing overall morbidity and mortality due to vascular complications [25].

Biliary complications following LT have been commonly viewed as the “Achilles heel” of the procedure, which have been reported in 10% to 29% of recipients [26,27]. Patients suffering from biliary complications have extended hospital stays, an increased incidence of major complications (such as HAT and T cell-mediated rejection), and higher comprehensive complication index scores [28]. In terms of the impact of biliary complications on graft and patient survival, Li et al. used biliary complications as the time-dependent variable in a Cox regression, revealing them as a risk factor for graft and patient survival [28]. In our study, biliary complications were also found to significantly predict graft survival in patients with BA, which matches the existing literature [29,30]. Although most can be successfully treated, biliary complications increase medical costs, diminish patient quality of life, and compromise long-term graft and patient survival [31]. Therefore, strategies, including careful donor selection, preoperative planning, expedited procurement techniques, and accumulated recipient surgical expertise, play a crucial role in reducing or eliminating the incidence and long-term consequences of this persistent Achilles heel of LT.

Despite the significance of this retrospective single-center study, there were several limitations. First, this was a single-center retrospective study, and the indications for different procedures were uncertain and inconsistent, thus inevitably leading to selection bias and limiting the generalizability to other centers. Second, the model was not externally validated, which might result in the overfitting of the model. Therefore, further in-depth multicenter studies with larger sample sizes are needed to verify the nomogram and risk classification system.

## 5. Conclusions

In conclusion, we have shown that a previous Kasai procedure has no impact on the patient or graft survival after LT in BA patients. Notably, the length of the ICU stay and bleeding complications after LT were the independent risk factors for postoperative patient mortality. A deceased donor, graft types other than whole liver and left lateral segment, vascular complications, and biliary complications were the independent prognostic factors for graft survival after LT in BA patients. Moreover, a nomogram and risk stratification system were further developed to predict the post-LT graft survival, and the comprehensive validation proved its excellent predictive and clinical application value.

## Figures and Tables

**Table 1:** Patient characteristics, with demographic and laboratory data, for all three subgroups.

Parameter	No Kasai, n = 151	Early Kasai, n = 287	Late Kasai, n = 37	*p*-Value	
Male, n (%)	71 (47.0)	145 (50.5)	23 (62.2)	0.25	
Age at Kasai, days	-	61 (51–72)	107 (95–118)	<0.001	
Weight, kg	7.3 (6.5–8.2)	9.3 (7.0–15.5)	8.5 (6.7–16.0)	<0.001	No vs. early KP: <0.001 No vs. late KP: 0.02
Age at LT, months	7.7 (6.2–9.5)	15.0 (8.5–46.9)	9.9 (7.8–56.4)	<0.001	No vs. early KP: <0.001 No vs. late KP: <0.001
ALT, U/L	340 (171–581)	317 (117–616)	321 (164–692)	0.43	
AST, U/L	530.4 (310.5–978.9)	491.7 (186.1–909.1)	398.5 (220.0–965.6)	0.18	
GGT, U/L	106 (29–237)	102 (46–201)	128 (48–349)	0.34	
TBIL, µmol/L	167.0 (96.2–261.3)	72.7 (39.1–139.2)	118.6 (46.9–204.4)	<0.001	No vs. early KP: <0.001 No vs. late KP: 0.04
WBC, ×10^9^/L	9.1 (6.4–13.3)	9.5 (6.1–13.4)	9.1 (6.4–12.8)	0.97	
Neutrophils %	58.3 (48.0–68.9)	63.8 (49.5–75.7)	68.8 (53.8–77.4)	0.003	No vs. early KP: 0.01 No vs. late KP: 0.02
RBC, ×10^12^/L	3.4 (3.0–3.9)	3.8 (3.2–4.2)	3.8 (3.0–4.4)	<0.001	No vs. early KP: <0.001
HB, g/L	100 (86–113)	103 (89–116)	101 (84–121)	0.58	
Platelet count, ×10^9^/L	132 (93–201)	129 (85–190)	125 (95–222)	0.49	

ALT, alanine aminotransferase; AST, aspartate aminotransferase; GGT, gamma-glutamyl transpeptidase; HB, hemoglobin; KP, Kasai hepatoportoenterostomy; LT, liver transplantation; RBC, red blood cell; TBIL, total bilirubin; WBC, white blood cell.

**Table 2:** Perioperative characteristics and postoperative complications for all three subgroups.

Parameter	No Kasai, n = 151	Early Kasai, n = 287	Late Kasai, n = 37	*p*-Value	
LT type				0.001	
LDLT	126 (83.4)	197 (68.6)	23 (62.2)		
DDLT	25 (16.6)	90 (31.4)	14 (37.8)		
GRWR, %	3.4 (3.0–4.0)	2.8 (2.1–3.6)	3.1 (2.1–4.0)	<0.001	No vs. early KP: <0.001 No vs. late KP: 0.03
Graft type				0.002	
Whole liver	23 (15.2)	81 (28.2)	12 (32.4)		
Left lateral segment	117 (77.5)	170 (59.2)	22 (59.5)		
Others	11 (7.3)	36 (12.5)	3 (8.1)		
Warm ischemia time, min	3.0 (2.0–5.0)	3.0 (2.0–5.0)	3.0 (3.0–5.0)	0.92	
Cold ischemia time, h	1.4 (1.1–2.1)	2.0 (1.4–6.0)	2.2 (1.5–6.3)	<0.001	No vs. early KP: <0.001 No vs. late KP: 0.002
Operation time, h	6.8 (5.8–7.5)	6.7 (5.8–7.6)	6.3 (5.4–7.5)	0.31	
Intraoperative blood loss, mL	240 (150–300)	200 (120–300)	160 (120–300)	0.23	
Duration of ventilation, h	3.0 (2.0–6.0)	3.0 (2.0–5.0)	3.0 (1.5–5.0)	0.03	No vs. early KP: 0.03
ICU stay, hours	92.0 (69.5–120.0)	78.3 (60.0–107.8)	87.0 (59.0–100.0)	<0.001	No vs. early KP: <0.001
Hospitalization, days	24 (19–35)	22 (17–32)	23 (18–30)	0.06	
Follow-up period, years	6.7 (4.0–8.5)	5.2 (3.2–6.6)	5.2 (3.0–6.6)	<0.001	No vs. early KP: <0.001 No vs. late KP: 0.004
Postoperative complications					
Vascular complications	8 (5.3)	5 (1.7)	1 (2.7)	0.11	
Biliary complications	3 (2.0)	19 (6.6)	4 (10.8)	0.03	
Infection	28 (18.5)	92 (32.1)	11 (29.7)	0.01	
Re-transplantation	1 (0.7)	4 (1.4)	1 (2.7)	0.40	
Bleeding	3 (2.0)	17 (5.9)	1 (2.7)	0.15	
Patient mortality	6 (4.0)	9 (3.1)	4 (10.8)	0.06	
Graft loss	7 (4.6)	13 (4.5)	5 (13.5)	0.04	

DDLT, deceased donor liver transplantation; GRWR, graft-to-recipient weight ratio; ICU, intensive care unit; LT, liver transplantation; LDLT, living donor liver transplantation; KP, Kasai hepatoportoenterostomy.

## Data Availability

The data presented in this study are available on request from the corresponding author due to ethical reason.

## Supplementary Materials

The following supporting information can be downloaded at: https://www.mdpi.com/article/10.3390/jcm14103328/s1. Table S1: Univariate and multivariate Cox regression analysis of associated factors with post-transplant patient mortality in BA patients. Table S2: Multivariate Cox regression analysis of associated factors with post-transplant graft loss in BA patients.

## Abbreviations

The following abbreviations are used in this manuscript:

BA	Biliary atresia
LT	Liver transplantation
KP	Kasai hepatoportoenterostomy
DCA	Decision curve analysis
GRWR	Graft-to-recipient weight ratio
CIT	Cold ischemic time
WIT	Warm ischemic time
ALT	Alanine aminotransferase
AST	Aspartate aminotransferase
GGT	Gamma-glutamyl transpeptidase
TB	Total bilirubin
SD	Standard deviation
IQR	Interquartile range
ROC	Receiver operating characteristic
WBC	White blood cell
HB	Hemoglobin
LDLT	Living donor liver transplantation
DDLT	Deceased donor liver transplantation
HAT	Hepatic artery thrombosis
PVT	Portal vein thrombosis
