# Supplementary material for: The Effect of a Kasai Procedure on Liver Transplantation in Children with Biliary Atresia: A Cohort Study
Source: J Clin Med. 2025 May 10;14(10):3328. doi: 10.3390/jcm14103328 (PMC12112731; doi:10.3390/jcm14103328)
Supplement: Supplementary file 1 [file jcm-14-03328-s001.zip › jcm-3589179-supplementary.pdf]

Supplementary Table S1. Univariate and multivariate Cox regression analysis of associated factors with post-transplant patient mortality in BA patients.

	Univariate			Multivariate		
	HR	95%CI	*P*	HR	95%CI	*p*
Group	3.04	1.18-7.82	0.02	2.11	0.89-5.02	0.09
Sex	2.03	0.37-10.98	0.41			
ALT, U/L	0.99	0.99-1.00	0.16			
AST, U/L	1.00	1.00-1.00	0.03	0.99	0.99-1.00	0.53
GGT, U/L	1.00	1.00-1.01	<0.001	1.00	0.99-1.00	0.54
TBIL, μmol/L	1.00	0.99-1.00	0.68			
Neutrophils%	1.05	1.00-1.10	0.03	0.99	0.96-1.02	0.34
Hb, g/L	9.80	0.93-1.03	0.44			
Platelet count, $\times 10^9$/L	9.99	0.90-1.01	0.83			
LT type	1.07	0.14-8.06	0.95			
GRWR %	0.33	0.18-0.61	<0.001	1.01	0.62-1.64	0.98
Graft type	0.20	0.07-0.54	0.002	1.25	0.55-2.88	0.59
Warm ischemia time, min	0.86	0.50-1.49	0.59			
Cold ischemia time, h	0.84	0.63-1.11	0.22			
Operation time, h	0.63	0.42-0.94	0.02	1.06	0.81-1.37	0.69
Intraoperative blood loss, mL	0.99	0.99-1.00	0.61			
ICU stay, hours	1.00	1.00-1.01	<0.001	1.00	1.00-1.00	0.002*
Vascular complications	0.03	0.00-0.26	0.002	3.85	0.70-21.16	0.12
Biliary complications	2.27	0.55-9.34	0.26			
Infection	2.06	0.26-16.35	0.50			
Bleeding	1.02	18.86-552.25	<0.001	5.37	1.58-18.24	0.007*

ALT, Alanine aminotransferase; AST, Aspartate aminotransferase; BA, Biliary atresia; GGT, Gamma-glutamyl transpeptidase; GRWR, Graft-to-recipient weight ratio; HB, Hemoglobin; HR, Hazard ratio; ICU, Intensive care unit; LT, Liver transplantation; TBIL, Total bilirubin.

Supplementary Table S2. Multivariate Cox regression analysis of associated factors with post-transplant graft loss in BA patients.

	Univariate			Multivariate		
	**HR**	**95%CI**	***p***	**HR**	**95%CI**	***p***
Group	3.38	1.75-6.53	**<0.001**	1.29	0.64-2.57	0.48
Sex	0.34	0.13-0.89	**0.03**	1.35	0.59-3.08	0.48
ALT, U/L	1.00	0.99-1.00	0.12			
AST, U/L	0.99	0.99-1.00	0.07			
GGT, U/L	0.99	0.99-1.00	0.64			
TBIL, μmol/L	0.99	0.99-1.00	0.28			
Neutrophils %	0.99	0.96-1.03	0.81			
Hb, g/L	0.99	0.96-1.01	0.29			
Platelet count, $\times10^9$/L	0.99	0.99-1.00	0.30			
LT type	5.56	1.72-17.97	**0.004**	6.20	2.22-17.38	**<0.001** *
GRWR %	1.42	0.77-2.60	0.26			
Graft type	3.03	1.53-6.01	**0.001**	2.03	1.11-3.72	**0.02** *
Warm ischemia time, min	1.34	0.99-1.83	0.06			
Cold ischemia time, h	1.05	0.88-1.24	0.61			
Operation time, h	1.38	1.04-1.83	**0.02**	0.98	0.75-1.30	0.91
Intraoperative blood loss, mL	1.00	1.00-1.00	**0.006**	1.00	0.99-1.00	0.06
ICU stay, hours	1.00	0.99-1.00	0.20			
Vascular complications	8.42	19.5-363.9	**<0.001**	5.60	1.78-17.60	**0.003** *
Biliary complications	3.48	1.12-10.82	**0.03**	4.55	1.22-16.95	**0.02** *
Infection	0.71	0.24-2.08	0.53			
Bleeding	1.76	0.29-10.77	0.54			

ALT, Alanine aminotransferase; AST, Aspartate aminotransferase; BA, Biliary atresia; GGT, Gamma-glutamyl transpeptidase; GRWR, Graft-to-recipient weight ratio; HB, Hemoglobin; HR, Hazard ratio; ICU, Intensive care unit; LT, Liver transplantation; TBIL, Total bilirubin.
*p<0.05
